# Genome architecture and the role of transcription

**DOI:** 10.1016/j.ceb.2010.03.004

**Published:** 2010-06

**Authors:** Argyris Papantonis, Peter R Cook

**Affiliations:** The Sir William Dunn School of Pathology, University of Oxford, South Parks Rd, Oxford OX1 3RE, United Kingdom

## Abstract

During development or in response to environmental stimuli, eukaryotic genes change both their expression and position in 3D nuclear space. Then, is a gene transcribed because of its position, or is position determined by transcription? Are genes stochastically or deterministically engaged in transcription cycles? Recent results confirm that RNA polymerases and their transcription factors play central roles in genome organization, and that stochastic events can give rise to apparently deterministic expression. As is so often the case in biology, structure both determines function and is influenced by it.

## Introduction

We now know that genomes are not folded randomly [1], but what are the major shaping forces? One key driver proves to be transcription of DNA (both coding and non-coding), deployed in four dimensions—space and time. Position locally along the chromatin fibre and globally in nuclear space affect transcriptional output, but is a gene transcribed because of its position or is position determined by transcription? Additionally, are genes stochastically or deterministically engaged in transcription cycles? Recent advances, made both genome-wide and on individual loci, provide some insights.

## Linear structure: walking down the genome

One might familiarize oneself with a city (an interphase nucleus) by walking down its roads (chromatin fibres), or wandering around its neighbourhoods (from fibre to fibre).

### The long and winding road

**Figure 1:**
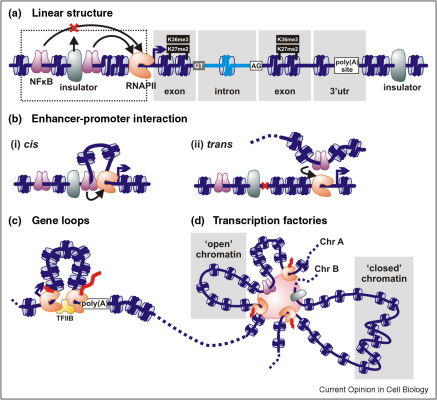
From linear to 3D architecture. (**a**) Linear structure. Genes typically encode an enhancer/promoter module (dotted outline) where RNA polymerases (RNAPII) and transcription factors dock (NFκB here), exons, introns and a 3′ untranslated region (utr); they are often flanked by insulators. Exons are nucleosome-rich and marked by H3K27-dimethyl and/or H3K36-trimethyl; splice sites (GT/AG) are nucleosome-poor; after the poly(A) site nucleosome density rises again. (**b**) Enhancer–promoter interactions deployed in *cis* (to generate a local loop) or *trans* may stimulate transcription. (**c**) Gene loop. The 5′ and 3′ ends of an active gene are juxtaposed, and tied by RNA polymerase and/or transcription factors (TFIIB here). (**d**) Transcription factories (pink) are polymorphic structures to which transcription units on the same or different chromosomes (Chr) are bound through RNA polymerases or transcription factors. ‘Open’ chromatin is transcribed when promoters in it attach to the factory; ‘closed’ chromatin is remote from the factory and inert [38].

A walk down the fibre would take you through alternating genic and non-genic regions (Figure 1a). A meta-analysis of deep-sequencing data shows that exons (compared to introns) in humans, mice, flies and worms are thickly populated with nucleosomes [2^•^,3^•^,4] and marked by higher H3K36 tri-methylation and H3K27 di-methylation [3^•^,4]; splice sites are unoccupied [2^•^]. Remarkably, these variations apply to both expressed and non-expressed genes. Additionally, poly(A) sites are nucleosome-depleted and followed by denser segments [4].

### Street features: polymerases, insulators and activators

We would often see RNA polymerase II along the way, and both genome-wide ‘run-ons’ and chromatin immunoprecipitation (ChIP) coupled to deep-sequencing now allow accurate localization of where the enzyme is [5^••^,6^••^]. Polymerases can be seen on ∼1/3 of human genes from promoters to beyond poly(A) sites, and on anti-sense strands at active promoters where they generate short divergent transcripts. The resulting transcriptional noise may be used by the cell to concentrate polymerase at promoters. In fact, in human T-cells, promoters of a subset of mitogen-responsive genes are ‘bookmarked’ by p300 and polymerases depleted of phospho-serine-2 and phospho-serine-5 in the C-terminal domain of the largest subunit. These promoters readily reactivate, and elongation factors reassemble on them upon addition of non-mitogenic agents that have minimal effects in the absence of preconditioning with mitogen [7]. Similarly, stalled polymerases on Drosophila *Hox* promoters can restart rapidly and serve as transcriptional insulators in concert with DSIF and NELF [8^•^]. RNAi-mediated NELF depletion stimulates transcription of 1/3 fly genes affected, presumably because a barrier to transcription is depleted; surprisingly, the rest of the affected genes are silenced, again presumably because they rely on stalled polymerases for efficient reactivation [9].

CTCF and its frequent partner, the cohesin subunit Rad21, mark boundaries (Figure 1a,b). They insulate enhancers from promoters [8], demarcate regions with distinct activities [10] and slow down transcribing polymerases [11]. For instance, the human apolipoprotein gene cluster is partitioned into two transcribed loops (detected using chromosome conformation capture, or 3C), and depleting either CTCF or Rad21 disrupts these to alter expression and binding of the transcriptional machinery [12]. In another example, bound Rad21 and CTCF mark imprinted loops in the interferon-γ locus [13].

Previously, transcriptional activators were thought to mark transcribed regions, but NFκB seems to use repeated *alu* elements throughout the genome as low-affinity ‘parking lots’. On virus infection, it translocates to the interferon-β enhancer to induce new inter-/intra-chromosomal loops and stimulate transcription of target genes [14,15].

### Heterochromatic suburbs

Heterochromatin is often peripheral. The striking exception of the rod nuclei of nocturnal mammals proves this rule: here, heterochromatin is central—an adaptation that channels more light to peripheral light receptors [16]. Heterochromatic genes are usually inactive, so is relocation to the periphery (or interior) sufficient to silence (or activate) a gene? Consistent with this, loci on six pig chromosomes became active and more internal during adipogenesis as their chromatin decondenses and ‘loops out’ from their respective territories (detected using FISH) [17]. Similarly, ChIP shows that ∼500 fly genes contact lamin B – presumably at the periphery – and these genes are clustered on the genetic map, quiescent and mid-to-late replicating; when coordinately activated during development, peripheral contacts are lost [18]. Again, genome-wide mapping shows >1300 human domains contact the lamina, and these are poorly expressed and have CTCF or CpG islands at their borders [19^•^]. This correlation of peripheral position and silencing was tested directly by tethering genes to the edge via lamin B [20] or *Lap2b*/emerin fusions [21]; tethering silenced some genes but not others. Moreover, mating type loci cluster during silencing independently of peripheral positioning [22].

### Limits to the effects of location

To what extent does location determine expression? ChIP-chip has revealed the tissue-specific pattern of binding of transcription factors to human chromosome 21, and – when this whole chromosome is transplanted into a mouse nucleus – this pattern remains essentially unchanged; clearly, DNA sequence is a major determinant of expression [23^•^]. Analogously, the locus control region (LCR) of human β-globin was inserted into a gene-dense and constitutively expressed region of the mouse genome; then, some genes up to 150 kb on each side were affected, with increased activity correlating with looping back to the LCR (detected by 3C; Figure 1b) [24].

## 3D structure: circular tours

Towns tend to have distinct financial, shopping, and residential zones. 3C coupled with deep-sequencing affirms that nuclei are also zoned. In yeast, contacts are non-randomly distributed, and – surprisingly – many are with mitochondrial and 2-micron plasmid DNA [25]. In man, a 1-Mbp resolution contact map confirms the presence of chromosome territories, the spatial proximity of gene-rich chromosomes and zoning into euchromatin and heterochromatin [26^••^]. Modeling reveals that such global positioning might well be driven by non-specific (entropic) forces, as well as ones like hydrogen bonds familiar to biologists [27].

### Round the block

Results from 3C and FISH substantiate the long-held view that enhancers contact target promoters both in *cis* [14,28–31] and *trans* [15] (Figure 1b). Interactions often correlate with transcriptional activation, perhaps involving scanning for partner elements [28]; in one case, exchanging GATA factors switches contacts and so alters gene expression [30]. Some promoters also contact 3′ ends of active genes to create gene loops (detected by 3C) (Figure 1c). In yeast, TFIIB and/or a component of the nuclear pore complex form the bridge [32–34]. In man, 5′ capping factors and RNA polymerases associate with 3′ end processing factors, again suggesting a gene loop forms; as such co-localization is enhanced by arresting elongation, polymerases at each end could recruit the processing machinery to facilitate production of the mature message [35].

### Active RNA polymerases at cross-roads

It is apparent from the above that bound RNA polymerases are often found at the cross-roads maintaining loops (as in Figure 1c,d); a genome-wide analysis confirms this. Human cells were stimulated with oestrogen, and contacts made by bound oestrogen receptor-α monitored by ChIP, 3C and deep sequencing; both contacting partners were often associated with bound RNA polymerase II [36^••^]—suggesting that polymerases might be the molecular ties maintaining loops.

### Factories in rotaries/roundabouts

Transcription factories are sites containing at least two (usually more) active transcription units [37,38]; a typical factory in the HeLa nucleoplasm contains ∼8 active templates and ∼8 nascent transcripts on the surface of a polymorphic protein-rich core (diameter ∼90 nm, mass ∼10 MDa) [39] (Figure 1d). These factories specialize in transcribing different sets of genes. For example, inserting an intron (or different promoter) into a mini-chromosome targets that mini-chromosome to a different ‘splicing’ (or promoter-specific) factory [40]; loci on 9 human chromosomes encoding cytochrome c oxidase (COX) subunits share the same ‘mitochondrial’ factories as genes on 3 other chromosomes encoding factors needed to transcribe mitochondria-encoded COX subunits [41]; and active haemoglobin-α and haemoglobin-β genes are found with other (active) erythropoiesis-related genes in ‘globin’ factories [42^•^].

## Temporal rhythms: the fourth dimension

In a city, different locales have their own temporal rhythms.

### Bursts of activity

**Figure 2:**
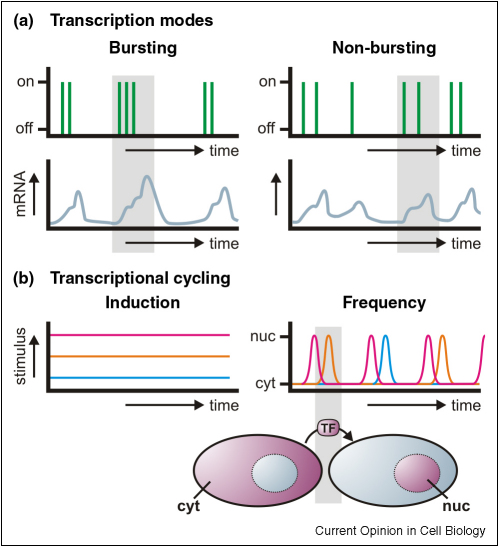
Temporal modes of gene expression. (**a**) Bursting. Genes may fire (stochastically) in tightly coordinate bursts, resulting in distinct peaks of mRNA (*left*); in non-bursting genes (e.g. constitutive), stochastic initiation yields more even mRNA levels (*right*). (**b**) Cycling. Different levels of agonist (*left*; blue < orange < red) affect the translocation frequency of the responding transcription factor (*right*). Cartoon: a cytoplasmic transcription factor (TF; purple) translocates to the nucleus in response to an agonist.

A developmentally controlled gene in Dictyostelium is transcribed in discrete pulses separated by irregular intervals, and this stochastic pulsing was more likely to recur than to initiate *de novo* [43] (Figure 2a). In Drosophila embryos, some developmentally controlled genes are transcribed stochastically in bursts, whilst others are expressed synchronously and uniformly—and this is associated with polymerase stalling [44]. In yeast, expression of tightly regulated genes varies substantially from cell to cell, whilst constitutively expressed ones exhibit less variation as single initiations tend to occur stochastically and not in bursts [45^•^].

### Repeated cycling

Adding a synthetic ligand to human embryonic kidney cells induces hourly cycles of activator binding, DNA looping and *PDK4* mRNA production (Figure 2b); modeling (using realistic concentrations and kinetic constants) shows that such cycling emerges simply from the intrinsic multi-step and irreversible nature of transcription [46]. In cells treated with short pulses of another agonist (TNFα), the activator (NFκB) cycles from cytoplasm to nucleus (Figure 2b). Higher frequency pulses reduce translocation, indicating a failure to reset the system, and deterministic/stochastic models involving feedback loops enable accurate prediction of the cycles [47^••^]. Cycling of GFP-p65 (an NFκB subunit) in living cells can also be modeled accurately by tuning the feedback loops [48]. The frequency (but not the duration) of cycling of another transcription factor – yeast Crz1 – is controlled by calcium concentration [49]. In all these cases, negative feedback loops coordinate the temporal rhythms.

A glance at a human genome browser reveals that many human genes are very long (>150 kb), and there may be method in such madness; transcribing a long gene can convert space into time. For example, tiling microarrays reveal that a pioneering polymerase takes more than an hour to transcribe a 220-kbp human gene switched on by TNFα. Polymerases that initiate subsequently on it soon abort if the pioneer is still transcribing; as a result, mature message is produced in one pulse after ∼1 h [11]. Introducing introns of different lengths into a synthetic reporter gene shows that increasing intron length can increase times between pulses [50], adding yet another checkpoint to the regulation of gene expression.

## Conclusions

Different city neighbourhoods may be filled with different sights, sounds and smells, but they usually have the same street features and general layout. Whereas ten years ago nuclei were only charted imprecisely, we are well on our way to mapping them at ever-increasing resolution. More and more of these maps are positioning RNA polymerases and their associated factors at important nodes in the genomic network (Figure 1b–d), so the transcription machinery determines structure—and structure inevitably influences function [38]. Although we can take pride in our maps, we should not delude ourselves. Nuclei are quite unlike cities—their structure changes from moment to moment and current high-throughput methods sample cell populations to yield data on an ‘average’ structure that probably never exists in any cell at any time. Nevertheless, evolution feeds from and works on the flexibility of genomic architecture; it constantly adapts to changing conditions to produce functional diversity. Then, there is no clear answer to both our questions (is a gene transcribed because of its position or is position determined by transcription, and are genes turned on/off stochastically or deterministically?) as structure determines function, and function inevitably alters the structure.

## Note

During the review process of this paper a new report was published describing the association of an inducible human gene (urokinase-type plasminogen activator) with specific transcription factories, before its activation by an external stimulus; RNA polymerases in these ‘poised’ factories lacked the characteristic phosphorylation of Ser2 of their CTD, indicative of efficient elongation activity [51]. Thus, yet another molecular tie – that between chromatin and ‘poised’ factories – seems to contribute to the architecture of eukaryotic genomes.

## References and recommended reading

Papers of particular interest, published within the annual period of review, have been highlighted as:

• of special interest
•• of outstanding interest
